# OMIP‐085: Cattle B‐cell phenotyping by an 8‐color panel

**DOI:** 10.1002/cyto.a.24683

**Published:** 2022-08-26

**Authors:** Eduard O. Roos, Marie Bonnet‐Di Placido, William N. Mwangi, Katy Moffat, Lindsay M. Fry, Ryan Waters, John A. Hammond

**Affiliations:** ^1^ The Pirbright Institute Surrey UK; ^2^ Animal Disease Research Unit, Agricultural Research Service US Department of Agriculture Pullman Washington USA; ^3^ Veterinary Microbiology and Pathology Department Washington State University Pullman Washington USA

**Keywords:** antibody secreting cells, B cells, B‐cell subsets, cattle, flow cytometry, naïve B cells, memory B cells, regulatory B cells

## Abstract

This 8‐color panel has been optimized to distinguish between functionally distinct subsets of cattle B cells in both fresh and cryopreserved peripheral blood mononuclear cells (PBMCs). Existing characterized antibodies against cell surface molecules (immunoglobulin light chain (S‐Ig[L]), CD20, CD21, CD40, CD71, and CD138) enabled the discrimination of 24 unique populations within the B‐cell population. This allows the identification of five putative functionally distinct B‐cell subsets critical to infection and vaccination responses: (1) naïve B cells (B_Naïve_), (2) regulatory B cells (B_Reg_), (3) memory B cells (B_Mem_), (4) plasmablasts (PB), and (5) plasma cells (PC). Although CD3 and CD8α can be included as an additional dump channel, it does not significantly improve the panel's ability to separate “classical” B cells. This panel will promote better characterization and tracking of B‐cell responses in cattle as well as other bovid species as the reagents are likely to cross react.

## 1 BACKGROUND

As our knowledge of immune cell subsets and their functions increases, so does the need to identify and measure alterations in their phenotype and frequency. The mammalian B‐cell population consists of several functionally distinct subsets that together comprise the major mediator of humeral immunity [1, 2]. The development of naïve B cells (B_Naïve_) is important for long‐term immune protection [3, 4, 5]. Driving the development of antibody secreting cells (ASC) and memory B cells (B_Mem_) is an essential requirement of many vaccines that elicit neutralizing antibody responses [6, 7, 8]. Furthermore, these subsets are often the source of therapeutic antibody candidates (as vaccines or immunotherapies) against infectious diseases [6, 7, 8]. Regulatory B cells (B_Reg_) also play a vital role in suppressing infectious diseases [9, 10]. Consequently, the identification and relative quantification of B‐cell subsets is a fundamental requirement when evaluating pathogen or vaccine‐induced immune responses and ultimately the development of better strategies to control diseases [1].

The capability to dissect B‐cell responses at high resolution is limited in many non‐model species through a combination of limited reagents, the lack of knowledge of species‐specific B‐cell markers and standardized methods [11, 12]. This is certainly the case for cattle, a key food producing species and crucial for human nutrition globally, as a universal B‐cell lineage marker (i.e., CD19) and reagents against other well‐known B‐cell subsets (e.g., IgD and CD38) are lacking [13]. As technologies to design and deliver protective immunogens continue to emerge rapidly, it is essential to evaluate their applicability in other species as part of one health approaches. Consequently, there is a need to study cattle B‐cell responses and their maturation at a high resolution.

We have developed a flow cytometry panel using the existing antibodies against cell surface markers based on knowledge in humans and mice [14] (Table 1). With no pan B‐cell markers known in cattle, such as CD19, CD72, or CD79α, we resolved B cells from other lymphocytes using CD14 (CCG33, [15]) to exclude the monocytes, CD40 (IL‐A158, [16]) as a B‐cell lineage marker, and included previously described cattle B‐cell markers such as CD21 (CC21, [17, 18]) and surface immunoglobulin light‐chain (S‐Ig(L), IL‐A58, [17, 19]) [2, 20] (Table 2). Subsets within these populations were further identified using the activation and differentiation markers CD71 (IL‐A165, [21]), CD20 (MEM‐97, [22]), and CD138 (recombinant‐F1.20/A, [Personal comm. Washington State University]).

**FIGURE 1:**
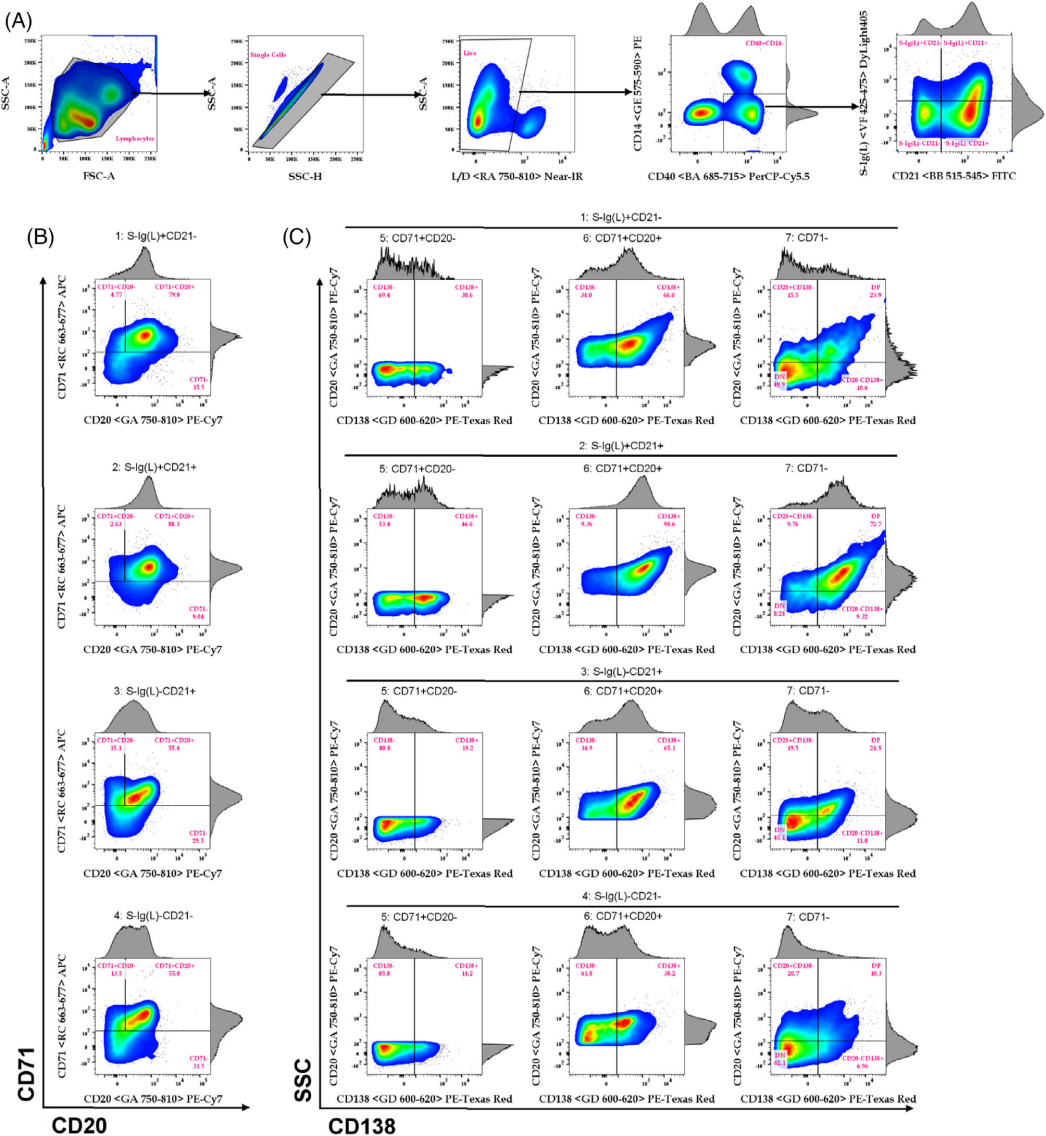
Gating strategy of the cattle B‐cell panel into 24 minor subsets. (A) the gating strategy followed from all events to the S‐Ig(L) vs CD21 populations. (B) S‐Ig(L) versus CD21 further sub‐gated into CD71 vs CD20 subsets for each of the four major subsets. (C) the 24 minor subsets identified as the CD138^+^ and CD138^−^ from the three previous subsets in B. the parent population for each of the 24 minor subsets are listed above each gated population [Color figure can be viewed at wileyonlinelibrary.com]

Based on well‐characterized human and mouse B‐cell populations, we hypothesize that these markers will identify five major subsets of B cells in cattle lymphocytes (Online Table 3): B_Naïve_, B_Mem_, B_Reg_, PB, and PC [14]. The panel further allows for more in‐depth characterization of cattle B cells into 24 phenotypically unique subsets, following the gating strategy set out in Figure 1; however, the functional discrimination and therefore importance between these subsets remains to be determined (Tables 1 and 2).

**TABLE 1:** Summary table for optimized multicolour immunofluorescence panel

Purpose	B‐cell phenotyping
Species	Cattle
Cell type	Fresh or cryopreserved PBMC
Cross reference	None

**TABLE 2:** Reagents used for optimized multicolour immunofluorescence panel

Specificity	Clone	Fluorochrome	Purpose
Ig(L)	IL‐A58	DyLight 405	B‐cell lineage, B‐cell subset
CD20	MEM‐97	PE‐Cy7	B‐cell development
CD21	CC21	FITC	B‐cell lineage, B‐cell subset
CD40	IL‐A158	PerCP‐Cy5.5	Co‐stimulatory, B‐cell lineage marker
CD71	IL‐A165	APC	Activation marker, activated B cells
CD138	r(F1.20/A)	PE‐Texas Red	Distinguishing of ASCs (PC vs. PB)
CD14	CCG33	PE	Dump, monocyte lineage marker
LIVE/DEAD	–	Near‐IR	Viability

Our gating strategy consists of plotting CD40 against CD14 to select “classical” (CD40^+^CD14^−^) B cells. Although CD3 and CD8α are often used as a dump channel to isolate cattle B cells, their inclusion did not significantly improve separation (Online Figure 8). After identifying B cells, S‐Ig(L) was plotted against CD21, allowing discrimination of four putative cattle B‐cell populations: CD21^−^S‐Ig(L)^+^and CD21^+^S‐Ig(L)^−^ single‐positive (SP), CD21^+^S‐Ig(L)^+^ double‐positive (DP), and CD21^−^S‐Ig(L)^−^ double‐negative (DN) cells (Figure 1 A. Next, each population was further sub‐divided by comparing CD71 against CD20 and sub‐gated into CD71^+^ CD20^−^ SP, CD71^+^CD20^+^ DP, and CD71^−^ populations (Figure 1B). Lastly, each of these sub‐gates were divided as either CD138^+^ or CD138^−^ (Figure 1 C), resulting in 24 minor subsets of cattle B cells. An important step while labeling the PBMCs is to first stain the cells with the CD20 antibody before adding any of the other antibodies in the panel (Online Figure 10).

By identifying functional subsets of B cells, this panel has the potential to dramatically improve our understanding of cattle immune responses to infection and vaccination, moving toward addressing some of the problems highlighted in both Entrican et al. and Barroso et al., for example, the lack of reagents to study the developmental cascade of cattle B cells [11, 13]. Additionally, the panel allows the enrichment or isolation of specific single B cells or their populations to further study function, specificity, and drive antibody discovery.

## AUTHOR CONTRIBUTIONS

**Eduard O. Roos:** Conceptualization (lead); data curation (lead); formal analysis (lead); investigation (lead); methodology (equal); project administration (lead); validation (equal); visualization (equal); writing – original draft (lead); writing – review & editing (equal). **Marie Bonnet‐Di Placido:** Data curation (equal); formal analysis (equal); investigation (supporting); methodology (supporting); supervision (equal); validation (equal); visualization (equal); writing – original draft (supporting); writing – review and editing (equal). **William N. Mwangi:** Formal analysis (supporting); methodology (supporting); resources (equal); supervision (supporting); writing – original draft (supporting); writing – review and editing (supporting). **Katy Moffat:** Data curation (supporting); formal analysis (supporting); methodology (supporting); resources (equal); software (lead); supervision (supporting); visualization (supporting); writing – review and editing (supporting). **Lindsay M. Fry:** Funding acquisition (supporting); resources (equal); writing – review and editing (supporting). **Ryan Waters:** Conceptualization (equal); funding acquisition (lead); investigation (supporting); methodology (supporting); project administration (supporting); resources (supporting); writing – original draft (supporting); writing – review and editing (supporting). **John A. Hammond:** Conceptualization (supporting); funding acquisition (supporting); methodology (supporting); project administration (supporting); resources (equal); supervision (lead); validation (supporting); writing – original draft (supporting); writing – review & editing (supporting).

## CONFLICT OF INTEREST

The authors have no conflict of interest to report.

## Supporting information


**Appendix S1** Supporting Information

## References

[1] Palm A‐KE , Henry C . Remembrance of things past: long‐term B cell memory after infection and vaccination. Front Immunol. 2019;10:1–13. 31417562 10.3389/fimmu.2019.01787 PMC6685390

[2] LeBien TW , Tedder TF . B lymphocytes: how they develop and function. Blood 2008;112:1570–80. Available from: https://ashpublications.org/blood/article/112/5/1570/25424/B-lymphocytes-how-they-develop-and-function. 18725575 10.1182/blood-2008-02-078071 PMC2518873

[3] Seifert M , Küppers R . Human memory B cells. Leukemia 2016;30:2283–92. Available from: http://www.nature.com/articles/leu2016226. 27499139 10.1038/leu.2016.226

[4] Kaminski DA , Wei C , Qian Y , Rosenberg AF , Sanz I . Advances in human B cell phenotypic profiling. Front Immunol. 2012;3:1–15. 10.3389/fimmu.2012.00302/abstract 23087687 PMC3467643

[5] Nutt SL , Hodgkin PD , Tarlinton DM , Corcoran LM . The generation of antibody‐secreting plasma cells. Nat Rev Immunol. 2015;15:160–71. 10.1038/nri3795 25698678

[6] Lambour J , Naranjo‐Gomez M , Piechaczyk M , Pelegrin M . Converting monoclonal antibody‐based immunotherapies from passive to active: bringing immune complexes into play. Emerg Microbes Infect. 2016;5:1, e92–9. 10.1038/emi.2016.97 PMC5034104 27530750

[7] Irani V , Guy AJ , Andrew D , Beeson JG , Ramsland PA , Richards JS . Molecular properties of human IgG subclasses and their implications for designing therapeutic monoclonal antibodies against infectious diseases. Mol Immunol. 2015;67:171–82. 10.1016/j.molimm.2015.03.255 25900877

[8] Bornholdt ZA , Turner HL , Murin CD , Li W , Sok D , Souders CA , et al. Isolation of potent neutralizing antibodies from a survivor of the 2014 Ebola virus outbreak. Science. 2016;351:1078–83. 10.1126/science.aad5788 26912366 PMC4900763

[9] Shen P , Fillatreau S . Suppressive functions of B cells in infectious diseases: Figure 1. Int Immunol. 2015;27:513–9. 10.1093/intimm/dxv037 26066008

[10] Rosser EC , Mauri C , Regulatory B . Cells: origin, phenotype, and function. Immunity. 2015;42:607–12. 10.1016/j.immuni.2015.04.005 25902480

[11] Entrican G , Lunney JK , Wattegedera SR , Mwangi W , Hope JC , Hammond JA . The veterinary immunological toolbox: past, present, and future. Front Immunol. 2020;11:1–8. 10.3389/fimmu.2020.01651/full 32849568 PMC7399100

[12] Mwangi W , Maccari G , Hope JC , Entrican G , Hammond JA . The UKveterinary immunological toolbox website: promoting vaccine research by facilitating communication and removing reagent barriers. Immunology. 2020;161:25–7. 10.1111/imm.13227 32548865 PMC7450168

[13] Barroso R , Morrison WI , Morrison LJ . Molecular dissection of the antibody response: opportunities and needs for application in cattle. Front Immunol. 2020;11:1–10. 10.3389/fimmu.2020.01175/full 32595642 PMC7304342

[14] Murphy K , Weaver C . Janeway's Immunobiology. In: Murphy K , Weaver C , editors. 9th ed. New York, NY: Garland Science/Taylor & Francis; 2018. p. 1–904.

[15] Sopp P , Kwong LS , Howard CJ . Identification of bovine CD14. Vet Immunol Immunopathol. 1996;52:323–8. 10.1016/0165-2427(96)05583-3 8896221

[16] Glew EJ , Carr BV , Brackenbury LS , Hope JC , Charleston B , Howard CJ . Differential effects of bovine viral diarrhoea virus on monocytes and dendritic cells. J Gen Virol. 2003;84:1771–80. 10.1099/vir.0.18964-0 12810871

[17] Naessens J , Newson J , McHugh N , Howard CJ , Parsons K , Jones B . Characterization of a bovine leucocyte differentiation antigen of 145,000 MW restricted to B lymphocytes. Immunology. 1990;69:525–30. 2185984 PMC1385623

[18] Naessens J , Hopkins J . Introduction and summary of workshop findings. Vet Immunol Immunopathol. 1996;52:213–35. 10.1016/0165-2427(96)05566-3 8896204

[19] Williams DJL , Newson J , Naessens J . Quantitation of bovine immunoglobulin isotypes and allotypes using monoclonal antibodies. Vet Immunol Immunopathol. 1990;24:267–83. 1692650 10.1016/0165-2427(90)90042-q

[20] Cyster JG , Allen CDC . B cell responses: cell interaction dynamics and decisions. Cell. 2019;177:524–40. 10.1016/j.cell.2019.03.016 31002794 PMC6538279

[21] Naessens J , Davis WC . Ruminant cluster CD71. Vet Immunol Immunopathol. 1996;52:257–8. 10.1016/0165-2427(96)05572-9 8896210

[22] Faldyna M , Samankova P , Leva L , Cerny J , Oujezdska J , Rehakova Z , et al. Cross‐reactive anti‐human monoclonal antibodies as a tool for B‐cell identification in dogs and pigs. Vet Immunol Immunopathol. 2007;119:56–62. 10.1016/j.vetimm.2007.06.022 17673300

